# Normal inflammation and regeneration of muscle following injury require osteopontin from both muscle and non-muscle cells

**DOI:** 10.1186/s13395-019-0190-5

**Published:** 2019-02-26

**Authors:** Dimuthu K. Wasgewatte Wijesinghe, Eleanor J. Mackie, Charles N. Pagel

**Affiliations:** Department of Veterinary Biosciences, Melbourne Veterinary School, Faculty of Veterinary and Agricultural Sciences, University of Melbourne, Parkville, Victoria 3010 Australia

**Keywords:** Osteopontin, Muscle injury, Inflammation, Regeneration, Macrophage, Neutrophil

## Abstract

**Background:**

Osteopontin is secreted by skeletal muscle myoblasts and macrophages, and its expression is upregulated in muscle following injury. Osteopontin is present in many different structural forms, which vary in their expression patterns and effects on cells. Using a whole muscle autograft model of muscle injury in mice, we have previously shown that inflammation and regeneration of muscle following injury are delayed by the absence of osteopontin. The current study was undertaken to determine whether muscle or non-muscle cells provide the source of osteopontin required for its role in muscle regeneration.

**Methods:**

The extensor digitorum longus muscle of wild-type and osteopontin-null mice was removed and returned to its bed in the same animal (autograft) or placed in the corresponding location in an animal of the opposite genotype (allograft). Grafts were harvested at various times after surgery and analysed by histology, flow cytometry and quantitative polymerase chain reaction. Data were analysed using one- or two-way ANOVA or Kruskal-Wallis test.

**Results:**

Immunohistochemistry confirmed that osteopontin was expressed by macrophages in osteopontin-null muscle allografts in wild-type hosts and by myoblasts in wild-type allografts in osteopontin-null hosts. The decrease in muscle fibre number observed in wild-type autografts following grafting and the subsequent appearance of regenerating fibres were delayed in both types of allografts to a similar extent as in osteopontin-null autografts. Infiltration of neutrophils, macrophages and M1 and M2 macrophage subtypes were also delayed by lack of osteopontin in the muscle and/or host. While the proportion of macrophages showing the M1 phenotype was not affected, the proportion showing the M2 phenotype was decreased by osteopontin deficiency. Expression of tumour necrosis factor-α and interleukin-4 was lower in osteopontin-null than in wild-type autografts, and there was no difference between the osteopontin-null graft types.

**Conclusions:**

Osteopontins from muscle and non-muscle sources are equally important in the acute response of muscle to injury, and cannot substitute for each other, suggesting that they play distinct roles in regulation of cell behaviour. Future studies of mechanisms of osteopontin’s roles in acute muscle inflammation and regeneration will need to investigate responses to osteopontins derived from both myoblasts and macrophages.

## Background

The response of muscle to injury involves infiltration of inflammatory cells, together with muscle fibre degeneration followed by fibre regeneration and restoration of normal muscle structure [1–3]. The inflammatory infiltrate includes phagocytic cells (neutrophils and macrophages), which assist in the removal of degenerating fibres; as this process is occurring, mononuclear myogenic precursor cells are activated to undergo proliferation, differentiation and fusion, forming new multinucleate muscle fibres or contributing to repair of existing damaged fibres.

The orderly sequence of events following muscle injury is regulated by many factors originating from both inflammatory and muscle cells. Included amongst these factors is osteopontin, a secreted phosphoprotein, which can be present in soluble form or immobilised in the extracellular matrix [2]. Osteopontin interacts with cells to influence their behaviour through receptors including CD44 and a variety of integrins [4]. Osteopontin is generally undetectable in normal mature muscle fibres; however, its expression is strongly upregulated in injured muscle, where it is expressed by macrophages and muscle cells [5–9]. In vitro, osteopontin supports the migration of neutrophils and macrophages, as well as adhesion, proliferation and differentiation of skeletal muscle myoblasts [6, 10, 11]. Using a whole muscle autograft model of acute muscle injury that involves disruption of innervation and the vascular supply, we have recently demonstrated that neutrophil and macrophage infiltration, and muscle fibre necrosis and regeneration are delayed in the absence of osteopontin [7].

While osteopontin exerts beneficial effects during the acute response to muscle injury, chronic overexpression of osteopontin in injured muscles appears to have negative consequences for muscle strength and function. Thus, in the chronically inflamed dystrophin-deficient muscles of mdx mice, osteopontin overexpression has been found to support fibrosis [12]. Furthermore, a single nucleotide polymorphism upstream of the transcriptional start site of the osteopontin (*SPP1*) gene has been identified as a strong genetic modifier of disease severity in Duchenne muscular dystrophy. Alleles of this SNP that result in increased osteopontin expression from muscle cells in response to glucocorticoid treatment have been associated with reduced muscle strength and decreased age to loss of ambulation [13, 14].

An explanation for these apparently contradictory effects of osteopontin in muscle may lie in its structural heterogeneity as well as the timing of its expression. Osteopontin is subject to alternative splicing and extensive posttranslational modifications including glycosylation, phosphorylation and sulphation, as well as further processing by cross-linking and proteolytic cleavage [2]. These various modifications influence osteopontin’s functional properties; for example, differentially phosphorylated forms exert different effects on cell adhesion [15], cleavage by thrombin reveals a cryptic integrin-binding site [16] and different isoforms differentially influence expression of cytokines in macrophages and myoblasts [17]. Moreover, different cell types secrete different isoforms and differentially modified forms of osteopontin [15, 18].

When undertaking further in vitro studies on osteopontin’s roles in muscle pathophysiology, it will be important to use the form/s of osteopontin to which the relevant cells are exposed in vivo. As a first step in this direction, we have undertaken a study aimed at determining which cellular source of osteopontin is required for normal acute responses to muscle injury. Here, we describe experiments involving whole muscle grafts in which extensor digitorum longus (EDL) muscles were allografted between wild-type mice and osteopontin-null mice, to determine whether the grafted muscle tissue or the host inflammatory infiltrate provides the necessary osteopontin. The responses in these two groups of mice were compared with those in wild-type and osteopontin-null mice that received autografts. We have also further characterised the nature of osteopontin’s role in the inflammatory response in injured muscle by examining M1 and M2 macrophage profiles and expression of inflammatory cytokines in the grafts.

## Materials and methods

### Animals

Osteopontin-null mice on a C57Bl/6J genetic background [19] were kindly provided by David Denhardt (Rutgers University, New Brunswick, NJ, USA). The colony was maintained by matings between heterozygous parents at the animal facility in the Melbourne Veterinary School, The University of Melbourne. Experimental animals (wild-type and osteopontin-null) were either littermates or the offspring of littermates. Mice were housed in a controlled environment with free access to food and water.

### Whole muscle grafting

**Fig. 1:**
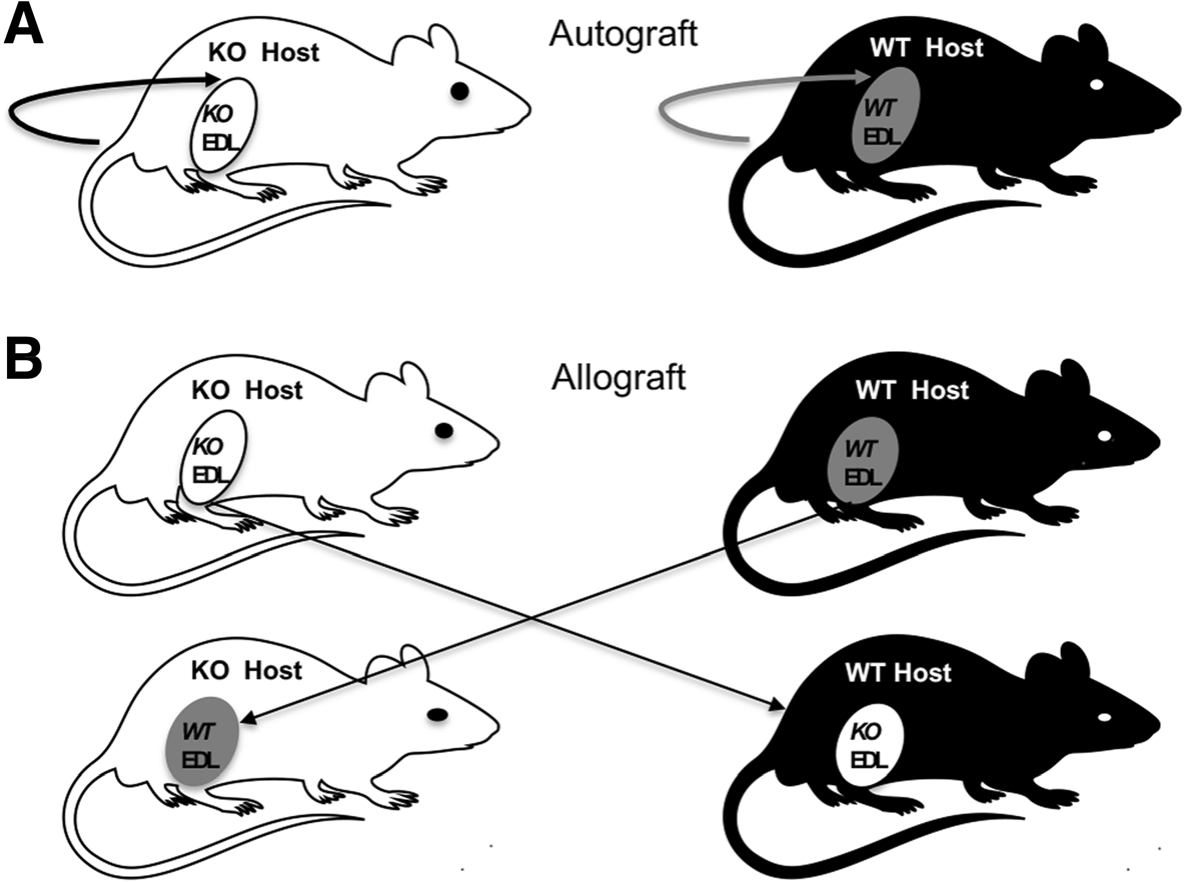
Schematic diagram of grafting procedures. **a** Autografting: the EDL muscles of osteopontin-null (KO; white) and wild-type (WT; black) mice were separated from the underlying muscle bed by incising the proximal and distal tendon of the EDL muscle and grafted onto the TA muscle of the same mouse. **b** Allografting: the EDL muscles of osteopontin-null and wild-type mice were removed from the right hind limb of the mice and grafted on the right TA muscle of the other genotype

Whole muscle grafting [20] was performed under aseptic conditions on anaesthetised 12-week-old male wild-type and osteopontin-null mice, which were randomly assigned to experimental groups. Briefly, a cutaneous incision was made over the right EDL and *tibialis anterior* (TA) muscles and the whole EDL muscle was excised from the adjacent muscle bed and connective tissue by incising the proximal and distal tendons of the EDL muscle. For autografting, the excised right EDL muscle was transplanted immediately over the underlying TA muscle by suturing the proximal and distal EDL tendons onto the proximal and distal ends, respectively, of the TA muscle using 5/0 coated Vicryl suture material (Johnson and Johnson Medical, North Ryde, NSW; Fig. 1a). Muscle allografting was performed in pairs of osteopontin-null and wild-type littermate mice. The right EDL muscles of each pair of mice were excised, and the donor EDL muscle of one genotype was transplanted onto the TA muscle of the host animal of the other genotype, and vice versa (Fig. 1b). As for autografting, the transplanted EDL muscle was sutured over the TA muscle. The cutaneous incision was then sutured closed, and analgesia was provided by injection of buprenorphine (Temgesic 100 μg/kg body weight). For all mice, the left TA muscle was used as a sham-operated control; the skin was incised and reflected from the muscle, then sutured closed. Animals were euthanized by CO_2_ inhalation on the 1st, 3rd, 5th, 7th and 14th days after the surgery, and the EDL muscles were harvested with the underlying TA muscle for histological analyses; EDL muscles were harvested alone from some animals for flow cytometry analysis or RNA extraction.

### T cell proliferation

Spleens were isolated from three pairs of allografted mice killed 14 days after grafting. Spleens were collected in FACS buffer comprised of phosphate-buffered saline (PBS) containing foetal calf serum (FCS; 2% *v*/*v*; Life Technologies, Waltham, MA, USA) and EDTA (5 mM) and disaggregated by passing through a 70-μm cell strainer; to lyse red blood cells, distilled water was added for 8 s prior to addition of PBS, then the cells were centrifuged and resuspended in RPMI 1640 medium (Life Technologies) containing 50 μM 2-mercaptoethanol, FCS (10% *v*/*v*), l-glutamine (2 mM), penicillin (100 U/ml) and streptomycin (10 U/ml; Pen-Strep-Glutamine; Life Technologies). Cells were plated in 96-well plates (2 × 10^6^ cells/well), then concanavalin A (Con A; 5 μg/ml), vehicle or osteopontin (0.005, 0.05, 0.5 or 5 μg/ml) was added to the wells. Plates were incubated for 72 h, then proliferation was assessed using 5-bromo-2′-deoxyuridine (BrdU) incorporation assay according to the manufacturer’s instructions (Roche BrdU Colorimetric Cell Proliferation ELISA; Roche Diagnostics Australia Pty. Ltd., Castle Hill, NSW, Australia); BrdU was present in the medium for the last 30 min of the incubation period.

### Histology and morphometric analyses

The harvested EDL and TA muscles were sectioned transversely in the mid belly region, and the muscle halves were embedded in OCT compound and frozen in isopentane chilled in liquid nitrogen. Serial transverse sections at the mid belly region of the EDL muscle were cut using a cryostat. Some sections were fixed in ethanol (70% *v*/*v*), stained using Carazzi’s haematoxylin and eosin (H&E) then mounted with DPX. Images of H&E-stained representative muscle sections were taken using SPOT Advanced Software (Diagnostic Instruments, Inc., Sterling Heights, MI, USA). The total muscle fibre numbers were counted on images of the whole EDL muscles using Adobe Photoshop CS5.1. Minimum Feret’s diameter of muscle fibres was measured as previously described [7].

### Immunohistochemistry

Representative muscle sections were fixed in paraformaldehyde (4% *w*/*v*) for 10 min at room temperature. After paraformaldehyde was washed off using PBS for 5 min at room temperature, the sections were blocked in FCS (5% *v*/*v* in PBS) for 30 min. Sections were then incubated for 1 h at room temperature with primary antibodies (Table 1), or non-immune serum or purified IgG diluted in PBS. Sections were washed three times with PBS for 5 min each. For unconjugated primary antibodies, sections were incubated with the fluorochrome-conjugated secondary antibody (Table 1) for 30 min at room temperature then washed three times in PBS. Sections were mounted in gelvatol [23% *v*/*v* polyvinyl alcohol, 50% *v*/*v* glycerol in PBS containing 0.1% *w*/*v* sodium azide (BDH) and 4′6-diamidino-2-phenylindole (DAPI; 1 μg/ml)]. Images of immuno-stained sections were captured under fluorescent illumination using SPOT Advanced Software.

**Table 1:** Antibodies used for immunohistochemistry

Primary antibody	Secondary antibody
FITC-conjugated rat anti-mouse Ly6G, Clone 1A8 (1:200; BD Biosciences, San Jose, CA, USA)	Not applicable
Rat anti-mouse F4/80 IgG2b Clone Cl:A3-1 (1:500; AbD Serotec, Oxford, UK)	Alexa Fluor 488-conjugated goat anti-rat IgG AB_2534074 (1:100; Abcam, Cambridge, MA, USA)
Goat anti-mouse osteopontin IgG ab11503 (10 μg/ml; R&D Systems, Minneapolis, MN, USA)	Alexa Fluor 594-conjugated donkey anti-goat IgG AB_2534105 (1:200; Abcam)
Mouse embryonic myosin heavy chain (F1.652.5; 2 μg/ml; Developmental Studies Hybridoma Bank, University of Iowa)	Alexa Fluor 488-conjugated goat anti-mouse IgG AB_2534069 (1:100; Life Technologies)
Mouse anti-desmin Clone DE-U-10 (1:80; Sigma-Aldrich St. Louis, MO, USA)	Alexa Fluor 488-conjugated goat anti-mouse IgG AB_2534069 (1:100; Life Technologies)
Rabbit anti-laminin L9393 (1:100; Sigma-Aldrich)	TRITC-conjugated swine anti-rabbit IgG A-11029 (1:100; Dako Cytomation, Glostrup, Denmark)

Neutrophils (stained with anti-Ly6G) were counted on images opened in Adobe Photoshop software. Cells were counted in nine specific fields (photographed using the × 20 objective) of each grafted EDL muscle, and the results were expressed as neutrophils per field. In sections stained with anti-embryonic myosin heavy chain (MyHC_emb_), anti-laminin and DAPI, small, centrally nucleated, MyHC_emb_-positive muscle fibres were counted as regenerating muscle fibres; results were expressed as regenerating muscle fibres per transverse section of grafted EDL muscle.

### Flow cytometry

Grafted EDL muscles were isolated 3, 5 and 7 days after grafting and incubated in PBS containing collagenase type 4 (20% *w*/*v*; Worthington, Lakewood, NJ, USA), dispase (0.025% *w*/*v*; Worthington) and CaCl_2_ (0.08% *w*/*v*) for 45 min at 37 °C. The digested muscles were mixed vigorously and passed through 100-μm and 40-μm filters then centrifuged at 400×*g* for 5 min. Red blood cells in the pellet were lysed as described above for spleen cells. The remaining cells were centrifuged and resuspended in 1 ml FACS buffer; viable cells were counted using the trypan blue exclusion method. Cells were incubated in FC blocker (WEHI Antibody Facility, Parkville, Victoria, Australia) for 5 min on ice then incubated in antibodies (all diluted 1:500) to F4/80 (allophycocyanin-conjugated rat anti-mouse F4/80; AbD Serotec), CD11c (phycoerythrin-Cy7-conjugated hamster anti-mouse CD11c; BD Biosciences) and CD206 (Alexa Fluor 488-conjugated rat anti-mouse CD206; BD Biosciences) on ice for 30 min. Cells were washed in FACS buffer, resuspended in FACS buffer and analysed in a FACSCANTO II analyser (BD Biosciences). Data were harvested using FACS Diva software (BD Biosciences) and analysed using FlowJo software (http://www.flowjo.com). Cells were gated from the debris using forward (FSC) and side (SSC) scatter, then single cells were gated using FSC width vs FSC area; from these, monocytes were identified as high FSC/low SSC. Macrophages were identified as F4/80-positive cells in the monocyte population, and M1 (CD11c-positive/CD206-negative) and M2 (CD206-positive/CD11c-negative) subsets were identified [21, 22]. Data are expressed as cells per grafted EDL muscle.

### RNA extraction and quantitative polymerase chain reaction

The EDL muscles from autografted and allografted wild-type and osteopontin-null mice were isolated 3, 5 and 7 days post-grafting. Purified and intact total RNA from the isolated EDL muscles were extracted using SV total RNA isolation system (Promega, Madison, WI, USA), according to the manufacturer’s instructions. First-strand complementary DNA (cDNA) was synthesised from extracted RNA using Go-script Reverse Transcription System (Promega) according to the manufacturer’s instructions.

The primers for osteopontin and interleukin-6 (IL-6) were as described [6, 23]. Primers for tumour necrosis factor-α (TNFα; forward 5' ACG GCA TGG ATC TCA AAG AC 3', 5"GTG GGT GAG GAG CAC GTA 3'), interleukin-4 (IL-4; forward 5′ TCG ATA AGC TGC ACC ATG AA 3′; reverse 5′ ATG ATG CTC TTT AGG CTT TCC A 3′) and the house-keeping gene cyclophilin A (forward 5′CAC AAA CGG TTC CCA GTT TT 3′; reverse 5′ TC ACC TTC CCA AAG ACC AC 3′) were designed using Primer 3 software (http://primer3.ut.ee/) and blastn; their specificity was confirmed by sequencing of PCR products. Cyclophilin A expression did not differ between experimental groups.

Quantitative polymerase chain reaction (qPCR) was performed using a Stratagene MX3000p Real-Time PCR Machine. A PCR reaction mixture was prepared using 10 μl SYBR Green PCR Master Mix (Life Technologies), 250 nM each forward and reverse primers (Geneworks, Hindmarsh, SA, Australia) and 1 μl cDNA template. The PCR reaction mixture was incubated using the following cycle profile: for amplification 95 °C for 10 min, followed by 40 cycles consisting of 95 °C for 20 s, 60 °C for 20 s and 72 °C for 20 s. The last cycle was 95 °C for 1 min, 70 °C for 30 s and 95 °C for 30 s. Fluorescence readings were acquired at the end of each extension step in the ROX/SYBR channel of the machine. After amplification, using the Ct values of the samples for each primer pair, the mean normalised expression (MNE; normalised to cyclophilin) of each gene was calculated using the Q-gene software [24].

### Statistical analysis

All data values are reported as the mean ± standard error (s.e.m.). Statistical analysis was performed using GraphPad Prism (GraphPad Software Inc., USA). In all cases, *p* < 0.05 was considered significant. For the BrdU incorporation assay, data were analysed using two-way ANOVA with Tukey’s multiple comparison test. For fibre and cell counts, data were analysed using one-way ANOVA with Tukey’s multiple comparison test. For qPCR data, comparisons between groups were performed using Kruskal-Wallis test with Dunn’s multiple comparison test.

## Results

### Investigation of immunological response to osteopontin in mice receiving muscle allografts

**Fig. 2:**
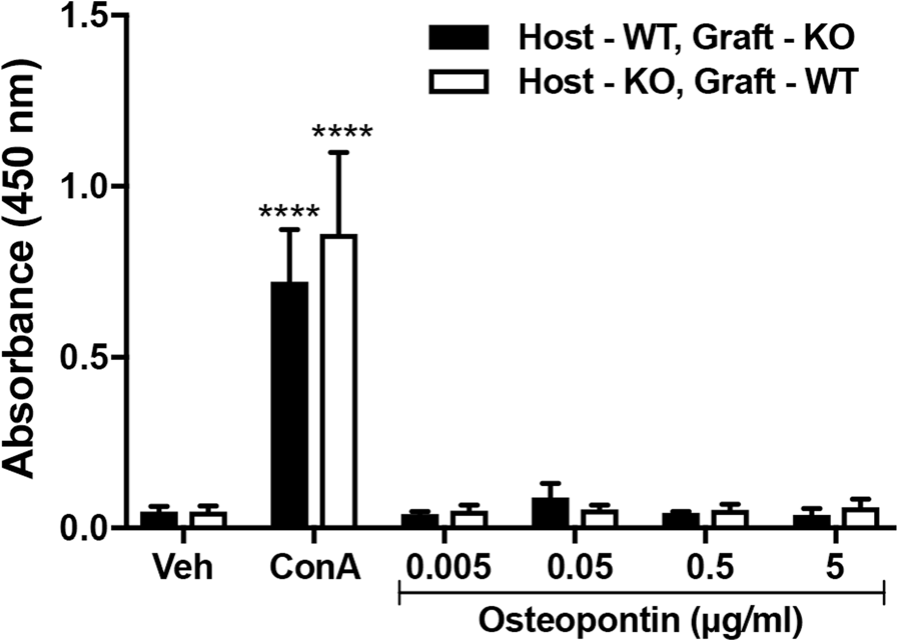
T cell proliferation in response to osteopontin. BrdU incorporation in spleen cells (isolated from mice that received allografts) treated with vehicle, Con A or recombinant mouse osteopontin. WT, wild-type; KO, osteopontin-null. Data are expressed as absorbance at 450 nm and presented as mean ± s.e.m.; *n* = 3 animals from each group. Two-way ANOVA with Tukey’s multiple comparison test was used to compare values with the value for vehicle-treated (Veh) cells of the same genotype; ****p < 0.0001

To rule out the possibility that wild-type EDL muscle grafts were rejected by osteopontin-null hosts, spleen cells were isolated from allografted wild-type and osteopontin-null hosts 14 days after grafting, and tested for proliferation in response to osteopontin. Whereas substantial BrdU incorporation occurred in cells from both genotypes in response to the positive control (Con A), there was no response to a range of osteopontin concentrations in cells from either genotype (Fig. 2), indicating that graft rejection had not occurred.

### Morphology of muscles grafted between wild-type and osteopontin-null mice

**Fig. 3:**
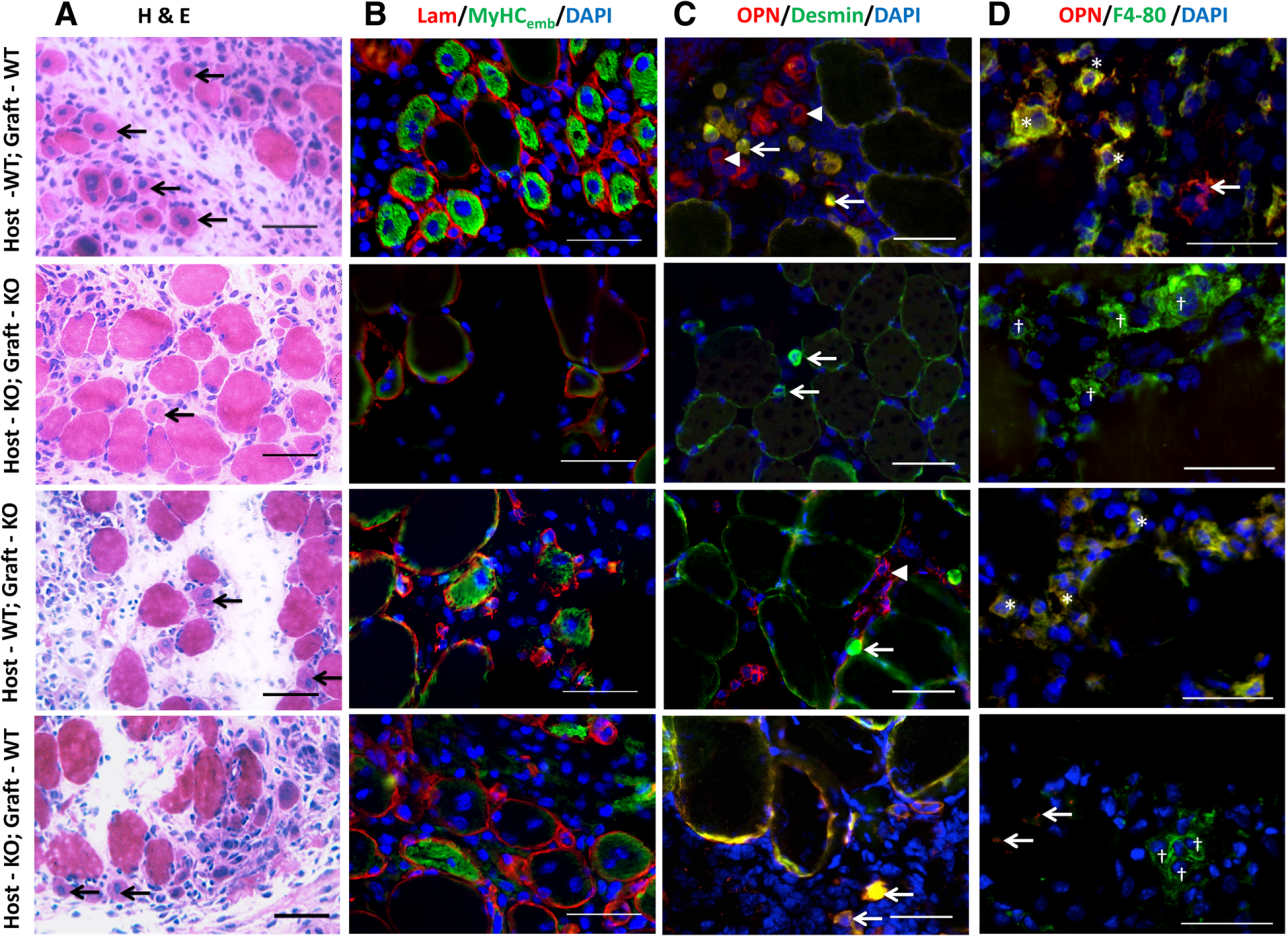
Autografted and allografted muscles: morphology and osteopontin expression. Cryosections of EDL muscle grafts at 5 (**a**, **b**) or 3 (**c**, **d**) days post-surgery. **a** H&E-stained sections. Arrows indicate regenerating fibres (small fibres with centrally located nuclei). **b** Sections stained with anti-MyHC_emb_ (green), anti-laminin (red) and DAPI, as used for quantitation of regenerating fibres. **c** Sections stained with anti-osteopontin (red), anti-desmin (myoblasts; green) and DAPI. In wild-type (WT) EDL muscle grafted into a wild-type host, anti-osteopontin stained both myoblasts (yellow) and non-myoblast mononuclear cells (red). In osteopontin-null (KO) muscle grafted into an osteopontin-null host, no osteopontin was detected. In allografted osteopontin-null muscle in a wild-type host, osteopontin was only detected in non-myoblast mononuclear cells. In allografted wild-type muscle in an osteopontin-null host, osteopontin was only detected in myoblasts. Arrows and arrowheads indicate myoblasts and non-myoblast mononuclear cells, respectively. **d** Sections stained with anti-osteopontin (red), anti-F4/80 (macrophages; green) and DAPI. In autografted wild-type muscle, anti-osteopontin stained macrophages (*; yellow) and some additional structures (arrows; red). In autografted osteopontin-null muscle, no osteopontin was detected. In allografted osteopontin-null muscle in a wild-type host, osteopontin was only observed in macrophages (*; yellow). In allografted wild-type muscle in an osteopontin-null host, weak osteopontin staining was observed in some non-macrophage structures (arrows), but not in macrophages (†; green). Bars = 50 μm

The morphology of EDL muscle grafts from autografted wild-type and osteopontin-null mice over the course of the experiment was as described [7]. In wild-type autografts, by day 3, there was substantial loss of muscle fibres and replacement by mononuclear cells including inflammatory cells. Centrally nucleated regenerating fibres were observed at day 5 (Fig. 3a) and appeared to increase in number over the ensuing days, replacing the mononuclear cells. By day 14, the muscles were again comprised primarily of muscle fibres. In osteopontin-null autografts, both the degeneration of necrotic fibres and appearance of regenerating fibres were delayed by about 2 days. The appearance of allografts in both wild-type and osteopontin-null hosts was similar to that of the osteopontin-null autografts at all time points (images of day 5 grafts shown in Fig. 3a).

**Fig. 4:**
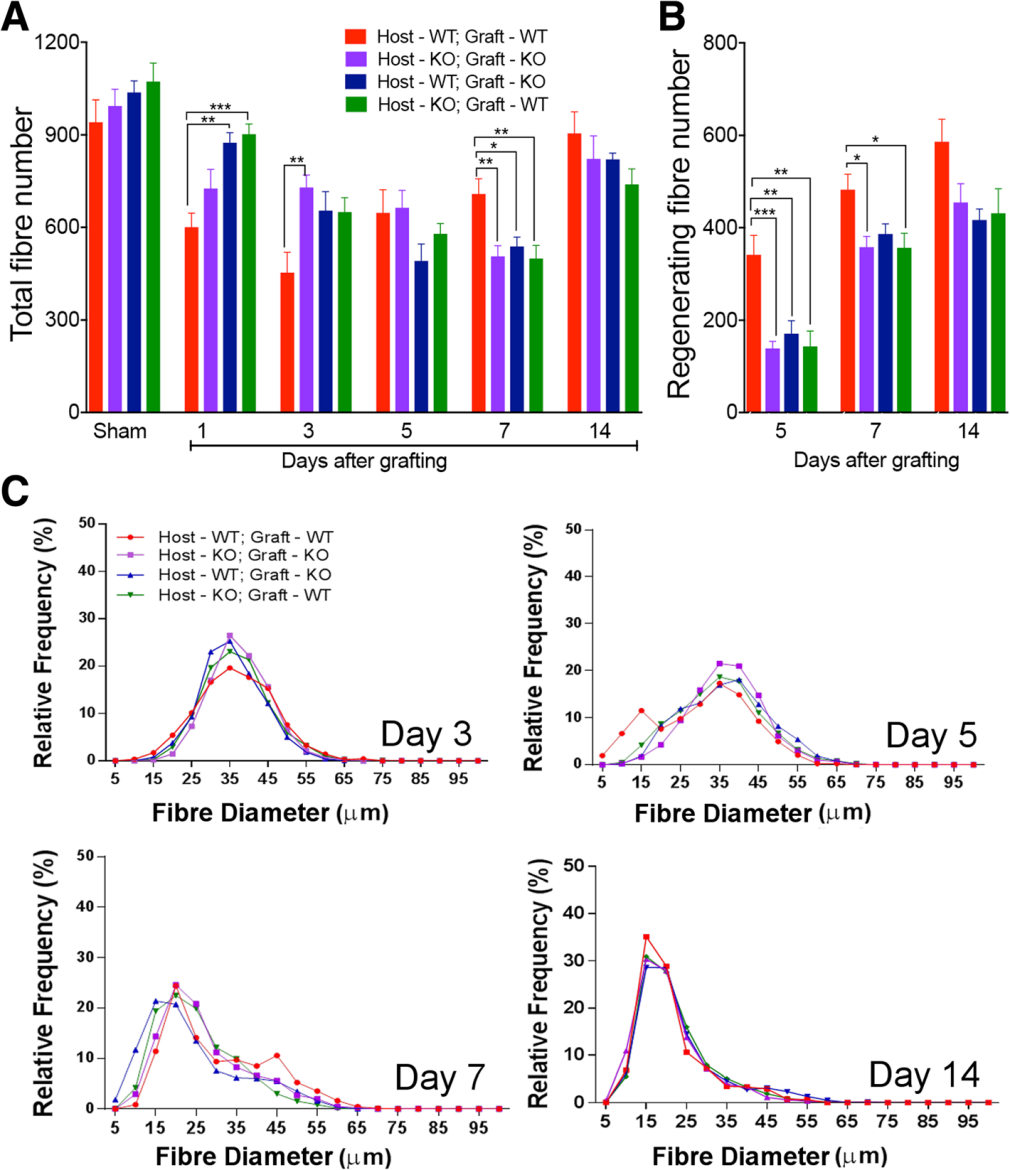
Histomorphometric analysis of grafted extensor digitorum longus muscles. **a** Total number of muscle fibres and **b** number of small, centrally nucleated, embryonic MyHC+ (regenerating) muscle fibres in sham-operated muscles at day 1 or in grafted EDL muscles at various times after grafting, expressed as number of muscle fibres per transverse section. WT, wild-type; KO, osteopontin-null. Data are presented as mean ± s.e.m. in *n* = 6 animals from each group at each time point. Data were analysed using one-way ANOVA with Tukey’s multiple comparison test. *p < 0.5, **p < 0.01 and ***p < 0.001. **c** Frequency histograms of minimum Ferret’s diameter of muscle fibres from autografted and cross-grafted muscles 3, 5, 7 and 14 days after surgery

The total number of muscle fibres per section was counted for each of the four types of muscle grafts at each time point, as well as for uninjured (sham-operated) muscles from each of the four groups at day 1 (Fig. 4a). One day post-injury, the fibre number in the autografted wild-type muscles was significantly lower than that in the contralateral uninjured muscles (*p* < 0.01). Significantly lower numbers of muscle fibres were present in autografted wild-type muscles than in both types of allografted muscles at this time point.

By 3 days post-grafting, the total fibre number in the wild-type autografted muscles was further reduced (*p* < 0.0001 in comparison with day 1 sham) and it was significantly lower in the osteopontin-null autografts (Fig. 4a). By day 5, the number of fibres in wild-type autografted muscles had started to increase, and there was no difference in the fibre number between groups. At this stage, regenerating muscle fibres had appeared as small diameter centrally nucleated MyHCemb-positive fibres in the grafts of all four experimental groups (Fig. 3b); however, there were significantly more regenerating fibres in the wild-type autografted muscles than in the other graft types (Fig. 4b).

At 7 days post-surgery, the total number of fibres was significantly greater in autografted wild-type muscles than in any of the other groups (Fig. 4a). Between day 5 and day 7, the numbers of regenerating muscle fibres increased in all the experimental groups (*p* < 0.05); however, they remained higher in the wild-type autografts than in osteopontin-null autografts or wild-type allografts in osteopontin-null host mice (Fig. 4b). The total muscle fibre numbers had increased in all groups 14 days after grafting to the point where they were close to the numbers observed in sham-operated muscles; no significant difference was observed between the four graft types (Fig. 4a). By this time, all the grafts consisted predominantly of regenerating muscle fibres (Fig. 4b).

At no time point was any difference observed in total or regenerating fibre number between graft types involving an osteopontin-null host or graft.

The Ferret’s minimal diameter of 300 fibres in each section were measured and plotted as a frequency histogram for each of the four types of muscle grafts at 3, 5, 7 and 14 days post-surgery (Fig. 4c). The distribution of minimal fibre sizes differed little between graft types 3 days post-grafting, with medians of 36.4 μm (wild-type autograft), 36.9 μm (osteopontin-null autograft), 34.9 μm (wild-type host) and 36.5 μm (osteopontin-null host). By 5 days post-grafting, a population of very small diameter fibres was apparent in wild-type autografted muscles (median 32 μm) but not in any of the other graft types (medians 36.8, 36.7 and 35.5 μm respectively). Smaller diameter muscle fibres predominated 7 days post-grafting (medians 20.2, 19.3, 15.4 and 17.7 μm respectively), suggesting by this time point most if not all fibres present were regenerating muscle fibres. The frequency distribution at day 14 was similar to that observed 7 days post-surgery, with more of the larger diameter fibres being replaced by smaller diameter fibres (medians 18.4, 18.6, 19.8 and 19.6 μm respectively).

### Expression of osteopontin in grafted muscles

Sections of the four types of muscle grafts harvested 3 days after grafting were immunostained with anti-osteopontin and anti-desmin to determine whether osteopontin expression was associated with myoblasts in allografted muscles (Fig. 3c). In wild-type autografts, osteopontin staining was observed in desmin-positive cells as well as in some desmin-negative mononuclear cells; in osteopontin-null autografts, no osteopontin staining was detected. In allografts in wild-type hosts, osteopontin was observed in desmin-negative mononuclear cells, but not in desmin-positive cells; in allografts in osteopontin-null hosts, the opposite was observed, that is, osteopontin was only detected in desmin-positive cells.

Sections were stained with anti-osteopontin and anti-F4/80 to determine whether osteopontin expression in allografts was associated with macrophages (Fig. 3d). In autografted wild-type muscles, anti-osteopontin stained macrophages as well as some other cells. In contrast, in the osteopontin-null grafts transplanted into wild-type hosts, osteopontin was only present in macrophages. In wild-type EDL muscles grafted into osteopontin-null mice, weak osteopontin staining was observed, but not in macrophages.

### Inflammatory cell infiltration in grafted muscles

In order to evaluate the efficiency of inflammatory cell infiltration into allografts following grafting, neutrophils and different phenotypes of macrophages were counted in the EDL muscle grafts. Grafted EDL muscles were too fragile to separate from the TA muscle at day 1, thus it was not possible to undertake flow cytometry at this stage. Since substantial numbers of neutrophils are already present in these grafts at day 1, immunohistochemistry was used for the detection of neutrophils.

**Fig. 5:**
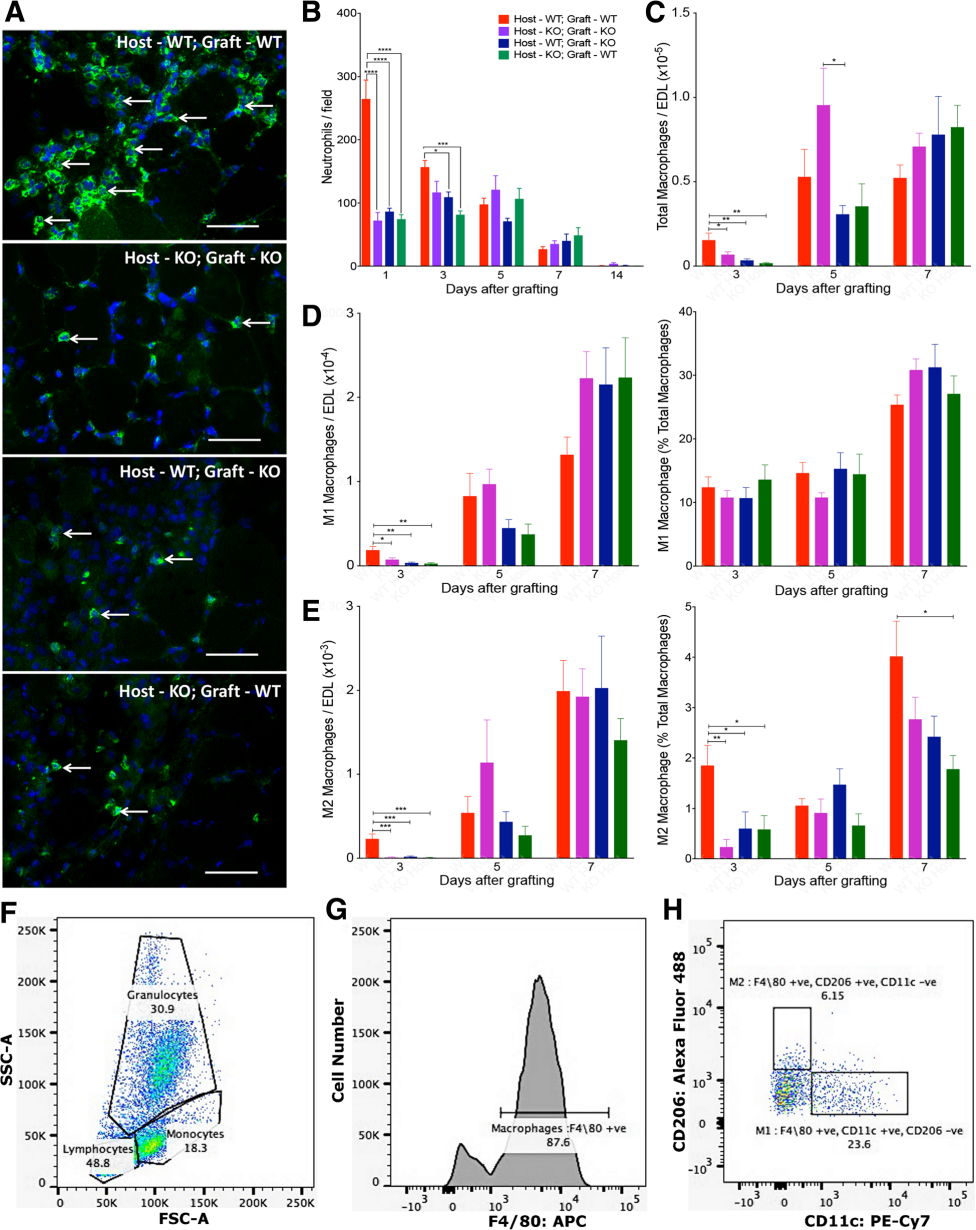
Inflammatory cells in autografted and allografted extensor digitorum longus muscles. **a** Representative transverse sections of EDL muscle grafts 1 day after grafting, immunostained with anti-Ly6G (green) and counterstained with DAPI (blue). Four combinations of wild-type (WT) and osteopontin-null (KO) host and graft. Arrows indicate neutrophils. Bar = 50 μm. **b** Quantitation of neutrophils in Ly6G-stained sections of grafts (expressed as neutrophils/field). **c**–**e** Quantitation of macrophages by flow cytometry. Numbers of total (F4/80-positive; **c**), M1 (**d**) and M2 (**e**) macrophages are expressed as cells/muscle, and M1 and M2 macrophage numbers are also expressed as a percentage of total macrophages (**d**, **e**). Data are presented as mean ± s.e.m. in *n* = 6 animals. Data were analysed using one-way ANOVA with Tukey’s multiple comparison test. *p < 0.05, **p < 0.01, ***p < 0.001 and ****p < 0.0001 for comparisons between graft types indicated by lines on graph. **f**–**h** Representative FACS scans showing gating of monocytes based on forward and side scatter (**f**), F4/80 staining (**g**) and CD206 and CD11c staining (**h**)

Within the first 24 h after transplantation, all four types of grafted EDL muscles contained neutrophils (Fig. 5a). On day 1, the number of neutrophils was significantly higher in wild-type autografted muscles compared to osteopontin-null autografted muscles and both wild-type and osteopontin-null allografts (Fig. 5b). By 3 days post-injury, the number of neutrophils in the autografted wild-type muscle had decreased compared to day 1; however, a significant difference between wild-type autografted muscles and the two allografted groups persisted. Over the subsequent days, neutrophil numbers gradually decreased in wild-type autografts, and there were no differences between groups after day 3.

Flow cytometry was used to quantitate total macrophage numbers as well as M1 and M2 subsets, which have previously been described as possessing inflammatory and regenerative phenotypes, respectively [3]. At day 3, the total macrophage number was significantly lower in osteopontin-null autografts and in both types of allografts than in wild-type autografts, and there were no significant differences between any of the three groups lacking osteopontin in host and/or graft (Fig. 5c). A small proportion of these cells had differentiated into M1 or M2 cells (~ 12% and ~ 2%, respectively, in wild-type autografts; Fig. 5d, e). The vast majority of the remaining F4/80-positive cells (~ 86%) were CD11c- and CD206-negative (Fig. 5h); presumably these cells were macrophages that had yet to fully polarise to either the M1 or M2 phenotype. For both of the fully polarised populations, there were significantly fewer cells in osteopontin-null autografts as well as in both types of allografts than in wild-type autografts. However, while the proportion of total macrophages that were classified as M1 (Fig. 5d) was unchanged between groups, the absence of osteopontin in host and/or muscle graft resulted in a significant decrease in the proportion of macrophages classified as M2 (Fig. 5e).

Macrophage numbers increased between day 3 and day 5 in all groups; at day 5, total, M1 and M2 macrophage numbers in all osteopontin-null groups were similar to those of wild-type autografts, although there were significantly fewer total macrophages in osteopontin-null allografts in wild-type hosts than in osteopontin-null autografts at day 5 (Fig. 5c–e). At day 7, there were no significant differences between groups, apart from a lower proportion of M2 macrophages in wild-type allografts in osteopontin-null hosts than in wild-type autografts (Fig. 5e).

### Gene expression in grafted muscles

**Fig. 6:**
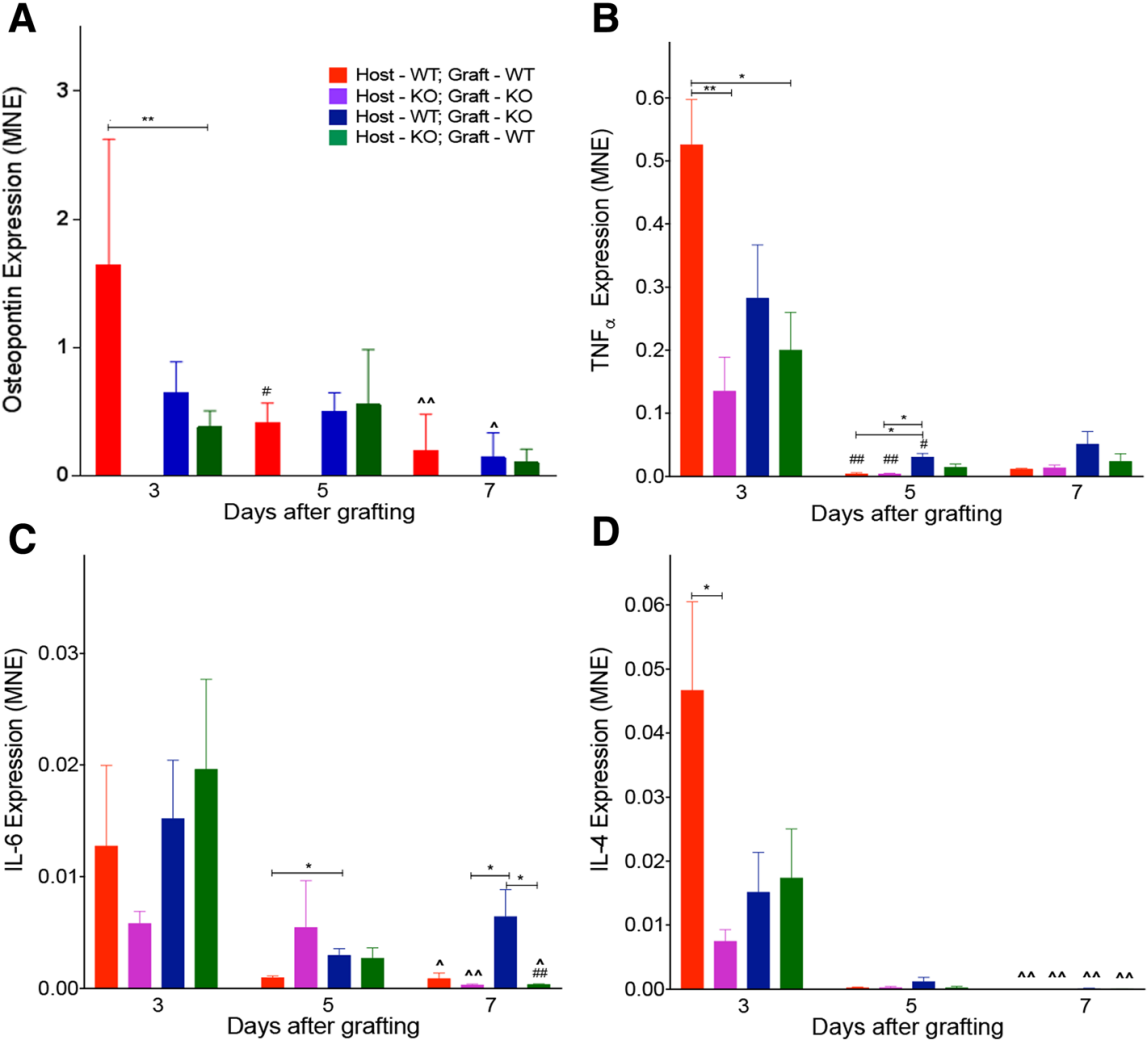
Osteopontin and inflammatory cytokines in autografted and allografted extensor digitorum longus muscles. Expression of osteopontin (**a**), TNFα (**b**), IL-6 (**c**) and IL-4 (**d**) in grafted muscles was assessed by qPCR

Expression of osteopontin mRNA was detected in wild-type autografts and in both types of allografts at day 3 post-surgery, but not in osteopontin-null autografts at any of the time points (Fig. 6a). Osteopontin expression was significantly higher in autografted wild-type muscle than in allografted wild-type muscle in osteopontin-null mice.

Expression of TNFα, IL-6 and IL-4 mRNA was detected in all four types of EDL muscle grafts 3 days post-surgery (Fig. 6b–d). At this time point, the expression of TNFα and IL-4 was significantly greater in wild-type autografted muscles than in osteopontin-null autografted muscles. The level of TNFα mRNA in autografted wild-type muscle was also significantly higher than in allografted wild-type muscle in osteopontin-null mice. However, there was no significant difference in TNFα or IL-4 levels between the two different types of allograft, and expression levels of both cytokines in allografts were similar to those of osteopontin-null autografts (Fig. 6b, d). The IL-6 expression 3 days following surgery in grafted EDL muscles was similar in the four experimental groups (Fig. 6c).

Between 3 and 5 days post-grafting, the levels of osteopontin in wild-type autografted muscles dropped significantly. Over the same period, TNFα in both types of autografted muscles and osteopontin-null EDL allografts in wild-type hosts also dropped significantly. At day 5, the TNFα expression in osteopontin-null muscle allografts in wild-type mice was higher than in both types of muscle autografts. The IL-6 expression in osteopontin-null allografted muscles was significantly higher than that in wild-type autografted muscles 5 days after grafting.

There was no significant difference in TNFα or IL-4 levels between different types of EDL muscle grafts at 7 days post-injury. There was also no difference in osteopontin expression between any of the grafts from a wild-type host and/or donor. Significantly greater expression of IL-6 was observed in osteopontin-null allografted muscles in wild-type hosts compared to both autografted osteopontin-null muscles and allografted wild-type muscles in osteopontin-null mice 7 days after surgery.

## Discussion

Osteopontin exists in many different structural forms, which vary between tissues and exert different effects on cells. The major aim of the current study was to determine whether the source of osteopontin required for osteopontin’s critical role in muscle regeneration is muscle or non-muscle cells, to inform future studies on biochemical mechanisms of this effect. The results of this study demonstrate that both of these sources are equally important in the early responses of muscle to injury induced by simultaneous denervation and disruption of the vascular supply. This observation applies to all osteopontin-dependent aspects of the muscle response to injury that we investigated, including the timing of muscle fibre degeneration, appearance of regenerating fibres, infiltration by neutrophils and macrophages (total, M1 and M2), and expression of TNFα and IL-4 mRNA.

The use of osteopontin-null mice in the whole muscle grafting model allowed us to distinguish between the roles of muscle-derived and non-muscle-derived osteopontin in the early response to injury. Previous studies have induced muscle injury by injecting myotoxic agents such as cardiotoxin [5]. This model induces muscle injury more rapidly than whole muscle grafting, but alone does not allow the contribution of host and donor genotype to muscle regeneration to be examined. The results of immunohistochemical studies presented here confirm our previous observations that desmin-positive (myogenic) cells express osteopontin and that this expression is upregulated following injury [6, 7]. They also confirm the observations of Hirata et al. that macrophages infiltrating injured muscles express osteopontin [5], and further suggest that macrophages are the major (if not the only) non-muscle source of osteopontin in whole muscle grafts 3 days post-surgery.

Following whole muscle autografting in wild-type mice, the number of muscle fibres initially decreases as they undergo necrosis and phagocytosis. From 5 days post-surgery, the fibre number starts to recover as a result of the presence of new regenerating fibres. We have previously shown that both the decrease and recovery of total fibre number are delayed by the absence of osteopontin, as is the appearance of regenerating fibres [7]. In the current study, while osteopontin-null autografts showed significantly higher fibre counts than wild-type autografts 2 days later than did the two types of allografts (day 3 as opposed to day 1), at neither of these time points was there any significant difference between the values for any of the three osteopontin-deficient graft types. Moreover, once substantial recovery of total fibre counts was observed in wild-type autografts (day 7), all three osteopontin-deficient graft groups showed significantly lower counts. Similarly, when regenerating fibres were first detected (day 5), there were significantly fewer in all of the osteopontin-deficient graft types than in the wild-type autografts, and there were no significant differences between any of the osteopontin-deficient graft types. Thus, it appears that both sources of osteopontin are required for both muscle fibre degeneration and regeneration, and they are not able to substitute for each other. This conclusion is further supported by the observation that while osteopontin mRNA expression was higher in wild-type autografts than in osteopontin-null allografts at day 3, there was no significant difference between allografts and wild-type autografts at later time points; this observation suggests that the source of the osteopontin is more important than the quantity present. Although osteopontin is known to be alternatively spliced and extensively post-translationally modified and processed, little is known about the forms of osteopontin expressed by myoblasts and macrophages. One possible explanation for the observed requirement for osteopontin from both sources for normal muscle regeneration is that the form of osteopontin secreted by muscle cells and inflammatory cells is different. Further work will be required to determine if this is the case.

The necrosis and phagocytosis of muscle fibres that precede fibre regeneration following injury are dependent on neutrophils and macrophages [25–27]. We have previously demonstrated that the initial infiltration by both of these cell types is dependent on osteopontin [7]. Here, we demonstrate that at the earliest time points examined for these cells (day 1 for neutrophils and day 3 for macrophages), counts for both cell types were significantly lower in all osteopontin-deficient graft types than in wild-type autografts, and the values for all the osteopontin-deficient graft types were similar to each other. Thus, infiltration of both neutrophils and macrophages is dependent on both muscle- and host-derived osteopontin. This requirement for both sources of osteopontin for phagocyte infiltration is likely to account for the fact that both sources are also required for the decrease in fibre number that normally occurs over the first few days following grafting.

Macrophages contribute not only to muscle fibre degeneration following injury, but also to regeneration of new fibres, through production of mediators that regulate myogenic precursor cell activity [3, 28]. In the current study, the delayed macrophage infiltration of grafts lacking one or both sources of osteopontin is therefore likely to contribute to the delayed appearance of regenerating fibres. However, it is likely that direct effects of osteopontin on myogenic precursor cells also contribute to regeneration through stimulation of proliferation and/or differentiation of these cells [6]. The results of the present study do not allow us to distinguish between indirect (macrophage-mediated) and direct effects of osteopontin on muscle regeneration, since macrophage infiltration was similarly delayed in all three osteopontin-deficient graft types.

In our earlier study of whole muscle autografts in osteopontin-null mice, we examined total (F4/80-positive) macrophages, but not macrophage subtypes [7]. Here, we were interested in determining whether osteopontin differentially influences the polarisation of these cells into either M1 macrophages, which are considered to be pro-inflammatory, or M2 macrophages, which are considered to be anti-inflammatory and pro-regenerative [3]. Both total M1 macrophage and total M2 macrophage counts were significantly reduced by osteopontin deficiency 3 days after grafting. All three osteopontin-deficient graft types showed similarly reduced values. The M1 and M2 macrophage counts in osteopontin-deficient grafts were returned to normal (i.e. similar to those of wild-type autografts) by 5 days after grafting. While the proportion of M1 macrophages was unaffected by osteopontin deficiency, at 3 days post-surgery the proportion of M2 macrophages was significantly lower in all osteopontin-deficient graft types than in wild-type autografts; these observations suggest that the deficiency in M1 macrophages is secondary to a requirement for osteopontin for infiltration of non-polarised macrophages, but that polarisation towards the M2 phenotype requires osteopontin. A recent study investigating the role of osteopontin in the chronic inflammation associated with dystrophin-deficient muscular dystrophy observed no effect of osteopontin ablation on the number of F4/80 macrophages in mdx muscle [29]. Our observation that total macrophage numbers in osteopontin-deficient grafts were no different from those of wild-type autografts by 5 days after initiation of acute inflammation are in keeping with this observation in chronically inflamed muscles. It is interesting to note that in the dystrophic muscles, osteopontin ablation resulted in a reduction in the proportions of M1 and M2a macrophage subtypes, and an increase in the proportion of the M2c macrophage subtype [29]. Our results with M1 and M2 macrophage populations may appear to contradict these observations; however, it is clear that osteopontin’s effects in muscle differ between acute and chronic inflammatory conditions (as noted in the Background). Moreover, we did not distinguish between M2a and M2c macrophage subtypes.

As part of our investigation of the effects of osteopontin deficiency on inflammation following acute muscle injury, we examined expression of mRNAs encoding three cytokines; the pro-inflammatory cytokines TNFα and IL-6 are associated with M1 macrophages, and IL-4 is associated with M2 macrophages [30]. At day 3, levels of both TNFα and IL-4 were lower in osteopontin-null autografts than in wild-type autografts, in keeping with the M1 and M2 macrophage counts; while the values for either one or both of the allografts were not significantly lower than those of wild-type autografts, they were also not significantly different from those of osteopontin-null autografts, so it cannot be concluded that the two sources of osteopontin exerted different effects. Expression of IL-6 at day 3 did not differ between groups; it is perhaps not surprising that IL-6 levels did not reflect macrophage numbers, since this cytokine is also expressed by muscle cells [31]. At the later time points, expression of IL-6 in allografts in wild-type hosts was higher than in wild-type autografts (day 5) or than in the other two osteopontin-deficient graft types (day 7), suggesting that for this one parameter, host-derived osteopontin may confer an advantage, perhaps even antagonising a suppressive effect of muscle-derived osteopontin.

## Conclusions

In vivo studies in mice indicate that osteopontin exerts contradictory effects in acute as compared with chronic inflammatory conditions of muscle, providing beneficial (pro-regenerative) effects in acutely inflamed muscle, but detrimental (pro-fibrotic) effects in chronically inflamed muscle [7, 12]. These differential effects are likely to be important in determining the outcome of various conditions involving muscle inflammation, but most particularly Duchenne muscular dystrophy, for which the osteopontin gene is a strong genetic modifier of disease severity [13]. We had hoped to identify either macrophage- or muscle-derived osteopontin as the key regulator of early inflammatory cell infiltration and/or muscle regeneration in acute muscle injury, which would have simplified the task of undertaking further studies on mechanisms of osteopontin’s effects on cells. However, the results presented here indicate that both sources are equally important, being unable to substitute for each other; both sources appear to play different, equally critical roles in the overall process of inflammation, muscle fibre degeneration, and regeneration following injury.

## Abbreviations

BrdU: 5-Bromo-2′-deoxyuridine
cDNA: Complementary DNA
DAPI: 4′6-Diamidino-2-phenylindole
EDL: Extensor digitorum longus
FCS: Foetal calf serum
H&E: Haematoxylin and eosin
IL-4: Interleukin-4
IL-6: Interleukin-6
KO: Osteopontin-null
MNE: Mean normalised expression
MyHC_emb_: Embryonic myosin heavy chain
PBS: Phosphate-buffered saline
qPCR: Quantitative polymerase chain reaction
TA: Tibialis anterior
TNFα: Tumour necrosis factor-α
WT: Wild-type

## References

[1] Sass FA, Fuchs M, Pumberger M, Geissler S, Duda GN, Perka C, Schmidt-Bleek K. Immunology guides skeletal muscle regeneration. Int J Mol Sci. 2018;19:1–19. 10.3390/ijms19030835 PMC5877696 29534011

[2] Pagel CN, Wasgewatte Wijesinghe DK, Taghavi Esfandouni N, Mackie EJ (2014). Osteopontin, inflammation and myogenesis: influencing regeneration, fibrosis and size of skeletal muscle. J Cell Commun Signal.

[3] Tidball JG (2017). Regulation of muscle growth and regeneration by the immune system. Nat Rev Immunol.

[4] Wang KX, Denhardt DT (2008). Osteopontin: role in immune regulation and stress responses. Cytokine Growth Factor Rev.

[5] Hirata A, Masuda S, Tamura T, Kai K, Ojima K, Fukase A, Motoyoshi K, Kamakura K, Miyagoe-Suzuki Y, Takeda S (2003). Expression profiling of cytokines and related genes in regenerating skeletal muscle after cardiotoxin injection: a role for osteopontin. Am J Pathol.

[6] Uaesoontrachoon K, Yoo HJ, Tudor EM, Pike RN, Mackie EJ, Pagel CN (2008). Osteopontin and skeletal muscle myoblasts: association with muscle regeneration and regulation of myoblast function in vitro. Int J Biochem Cell Biol.

[7] Uaesoontrachoon K, Wasgewatte Wijesinghe DK, Mackie EJ, Pagel CN (2013). Osteopontin deficiency delays inflammatory infiltration and the onset of muscle regeneration in a mouse model of muscle injury. Dis Model Mech.

[8] Barbosa-Souza V, Contin DK, Filho WB, de Araujo AL, Irazusta SP, da Cruz-Hofling MA (2011). Osteopontin, a chemotactic protein with cytokine-like properties, is up-regulated in muscle injury caused by Bothrops lanceolatus (fer-de-lance) snake venom. Toxicon.

[9] Hoffman EP, Gordish-Dressman H, McLane VD, Devaney JM, Thompson PD, Visich P, Gordon PM, Pescatello LS, Zoeller RF, Moyna NM (2013). Alterations in osteopontin modify muscle size in females in both humans and mice. Med Sci Sports Exerc.

[10] Giachelli CM, Lombardi D, Johnson RJ, Murry CE, Almeida M (1998). Evidence for a role of osteopontin in macrophage infiltration in response to pathological stimuli in vivo. Am J Pathol.

[11] Koh A, da Silva AP, Bansal AK, Bansal M, Sun C, Lee H, Glogauer M, Sodek J, Zohar R (2007). Role of osteopontin in neutrophil function. Immunology.

[12] Vetrone SA, Montecino-Rodriguez E, Kudryashova E, Kramerova I, Hoffman EP, Liu SD, Miceli MC, Spencer MJ (2009). Osteopontin promotes fibrosis in dystrophic mouse muscle by modulating immune cell subsets and intramuscular TGF-beta. J Clin Invest.

[13] Pegoraro E, Hoffman EP, Piva L, Gavassini BF, Cagnin S, Ermani M, Bello L, Soraru G, Pacchioni B, Bonifati MD (2011). SPP1 genotype is a determinant of disease severity in Duchenne muscular dystrophy. Neurology.

[14] Vianello S, Pantic B, Fusto A, Bello L, Galletta E, Borgia D, Gavassini BF, Semplicini C, Soraru G, Vitiello L, Pegoraro E (2017). SPP1 genotype and glucocorticoid treatment modify osteopontin expression in Duchenne muscular dystrophy cells. Hum Mol Genet.

[15] Christensen B, Kazanecki CC, Petersen TE, Rittling SR, Denhardt DT, Sorensen ES (2007). Cell type-specific post-translational modifications of mouse osteopontin are associated with different adhesive properties. J Biol Chem.

[16] Yokosaki Y, Matsuura N, Sasaki T, Murakami I, Schneider H, Higashiyama S, Saitoh Y, Yamakido M, Taooka Y, Sheppard D (1999). The integrin alpha(9)beta(1) binds to a novel recognition sequence (SVVYGLR) in the thrombin-cleaved amino-terminal fragment of osteopontin. J Biol Chem.

[17] Many GM, Yokosaki Y, Uaesoontrachoon K, Nghiem PP, Bello L, Dadgar S, Yin Y, Damsker JM, Cohen HB, Kornegay JN (2016). OPN-a induces muscle inflammation by increasing recruitment and activation of pro-inflammatory macrophages. Exp Physiol.

[18] Gimba ER, Tilli TM (2013). Human osteopontin splicing isoforms: known roles, potential clinical applications and activated signaling pathways. Cancer Lett.

[19] Rittling SR, Matsumoto HN, McKee MD, Nanci A, An XR, Novick KE, Kowalski AJ, Noda M, Denhardt DT (1998). Mice lacking osteopontin show normal development and bone structure but display altered osteoclast formation in vitro. J Bone Miner Res.

[20] Roberts P, McGeachie JK (1990). Endothelial cell activation during angiogenesis in freely transplanted skeletal muscles in mice and its relationship to the onset of myogenesis. J Anat.

[21] Fujisaka S, Usui I, Bukhari A, Ikutani M, Oya T, Kanatani Y, Tsuneyama K, Nagai Y, Takatsu K, Urakaze M (2009). Regulatory mechanisms for adipose tissue M1 and M2 macrophages in diet-induced obese mice. Diabetes.

[22] Ono Y, Nagai M, Yoshino O, Koga K, Nawaz A, Hatta H, Nishizono H, Izumi G, Nakashima A, Imura J (2018). CD11c+ M1-like macrophages (MPhis) but not CD206+ M2-like MPhi are involved in folliculogenesis in mice ovary. Sci Rep.

[23] Francis N, Ayodele BA, O'Brien-Simpson NM, Birchmeier W, Pike RN, Pagel CN, Mackie EJ (2018). Keratinocyte-specific ablation of protease-activated receptor 2 prevents gingival inflammation and bone loss in a mouse model of periodontal disease. Cell Microbiol.

[24] Muller P, Janovjak H, Miserez A, Dobbie Z (2002). Processing of gene expression data generated by quantitative real-time RT-PCR. BioTechniques.

[25] Nguyen HX, Tidball JG (2003). Interactions between neutrophils and macrophages promote macrophage killing of rat muscle cells in vitro. J Physiol.

[26] Hodgetts S, Radley H, Davies M, Grounds MD (2006). Reduced necrosis of dystrophic muscle by depletion of host neutrophils, or blocking TNFalpha function with Etanercept in mdx mice. Neuromuscul Disord.

[27] Pizza FX, Hernandez IJ, Tidball JG (1998). Nitric oxide synthase inhibition reduces muscle inflammation and necrosis in modified muscle use. J Leukoc Biol.

[28] Lescaudron L, Peltekian E, Fontaine-Perus J, Paulin D, Zampieri M, Garcia L, Parrish E (1999). Blood borne macrophages are essential for the triggering of muscle regeneration following muscle transplant. Neuromuscul Disord.

[29] Capote J, Kramerova I, Martinez L, Vetrone S, Barton ER, Sweeney HL, Miceli MC, Spencer MJ (2016). Osteopontin ablation ameliorates muscular dystrophy by shifting macrophages to a pro-regenerative phenotype. J Cell Biol.

[30] Locati M, Mantovani A, Sica A (2013). Macrophage activation and polarization as an adaptive component of innate immunity. Adv Immunol.

[31] Kami K, Senba E (1998). Localization of leukemia inhibitory factor and interleukin-6 messenger ribonucleic acids in regenerating rat skeletal muscle. Muscle Nerve.

